# DNA-based control of protein activity

**DOI:** 10.1039/c5cc09853j

**Published:** 2016-01-26

**Authors:** W. Engelen, B. M. G. Janssen, M. Merkx

**Affiliations:** a Laboratory of Chemical Biology and Institute for Complex Molecular Systems Eindhoven , University of Technology , Den Dolech 2 , 5600 MB Eindhoven , The Netherlands . Email: m.merkx@tue.nl

## Abstract

DNA has emerged as a highly versatile construction material for nanometer-sized structures and sophisticated molecular machines and circuits. The successful application of nucleic acid based systems greatly relies on their ability to autonomously sense and act on their environment. In this feature article, the development of DNA-based strategies to dynamically control protein activity *via* oligonucleotide triggers is discussed. Depending on the desired application, protein activity can be controlled by directly conjugating them to an oligonucleotide handle, or expressing them as a fusion protein with DNA binding motifs. To control proteins without modifying them chemically or genetically, multivalent ligands and aptamers that reversibly inhibit their function provide valuable tools to regulate proteins in a noncovalent manner. The goal of this feature article is to give an overview of strategies developed to control protein activity *via* oligonucleotide-based triggers, as well as hurdles yet to be taken to obtain fully autonomous systems that interrogate, process and act on their environments by means of DNA-based protein control.

## Abstract 2

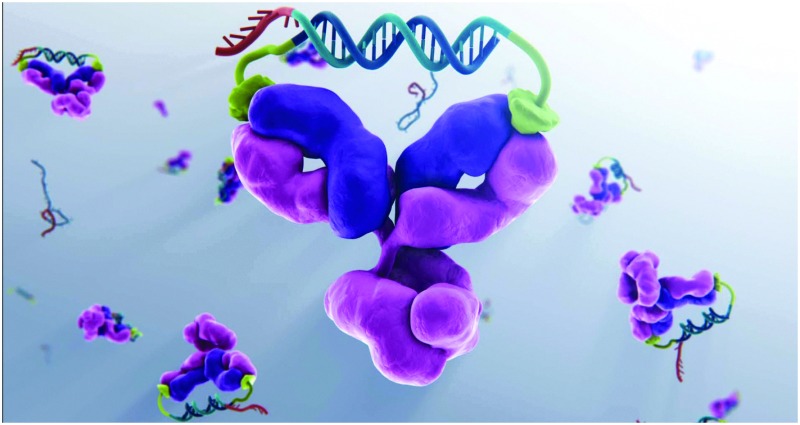
This feature article discusses the development of generic strategies to dynamically control protein activity *via* DNA-based triggers.

## 1. Introduction

Deoxyribonucleic acid (DNA) is best known as the blueprint of life, directing the synthesis of ribonucleic acid (RNA) and thus subsequent protein synthesis. Besides carrying our genetic information, DNA has proven to be a very versatile molecular building block in nanotechnology. Two fields of applications for DNA–nanotechnology can be distinguished: (1) structural DNA–nanotechnology, where oligonucleotides are used as a construction material to build precisely-defined nanometer-sized structures and (2) DNA-based molecular computing using DNA as a dynamic information carrier. Because of its inherent compatibility with biological systems, biomedical applications of DNA–nanotechnology are within reach. For such applications to become a reality, DNA-based systems need to be able to sense, process information and control their environments. One approach to increase the ‘functionality’ of DNA–nanotechnology has been to develop DNA-based alternatives for molecular functions that are typically performed by proteins, such as aptamers (ligand binding) and DNAzymes (catalysis).^1,2^ However, the number of functions provided by these DNA-based substitutes is still limited and does not rival those offered by proteins. Therefore, generally applicable strategies are required that allow oligonucleotide-based control of protein activity. The aim of this feature article is to provide an overview of molecular approaches that have been developed for oligonucleotide-based control of protein activity. Before the various strategies are discussed, we first provide some background on the molecular properties of DNA and the molecular principles that are employed in structural DNA–nanotechnology and DNA-based computing.

## 2. DNA as a molecular building block for 3D nanostructures and molecular computing

**Fig. 1:**
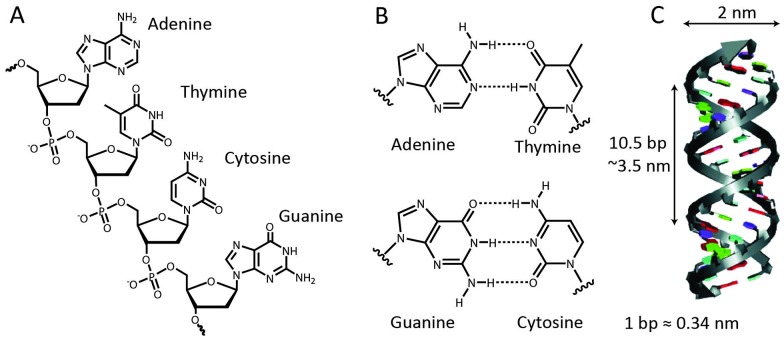
Structural properties of nucleic acids. (A) Adenine, guanine, thymine and cytosine bases are connected to the sugar-phosphate backbone *via* the 1′ sugar carbon. (B) Watson–Crick base pairing: complementary adenine and thymine bases form two hydrogen bonds, whereas guanine and cytosine bases form three hydrogen bonds. (C) π–π stacking interactions between the aromatic nucleobases result in the formation of a stable double-stranded helix.

In biology, DNA is generally present in a double-stranded helical form, with both strands running in an opposite, antiparallel orientation. The backbone of each DNA strand consists of alternating deoxyribose sugars and phosphate groups that connect the 5′ carbon of the deoxyribose sugar to the 3′ carbon of the subsequent sugar. Connected to the 1′ carbon of the sugars are the nucleobases that provide the quaternary code used for storage of genetic information (Fig. 1A). The four nucleobases exhibit a distinct hydrogen bond pattern where purines (adenine and guanine) interact with pyrimidines (thymine and cytosine, respectively). These hydrogen bonds, known as Watson–Crick base pairs, make DNA hybridization between two complementary single-stranded DNA polymers highly predictable (Fig. 1B). Although Watson–Crick base pairing provides selectivity, thermodynamic stability predominantly relies on π–π stack interactions between the aromatic nucleobases. The most common form of DNA is the B-type double helix with a diameter of 2 nm and a helical periodicity of 10.5 base pairs per turn (∼3.5 nm).^3^ Double stranded DNA can be considered as a rigid rod with a persistence length of 50 nm, while single stranded DNA resembles a flexible polymer chain with a persistence length of ∼1 nm.^4^ We will see below that this difference in mechanical properties has been extensively exploited to control protein activity.

**Fig. 2:**
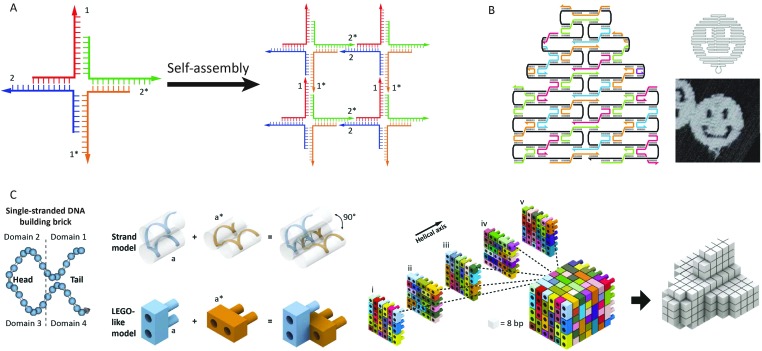
Advances in structural DNA–nanotechnology. (A) Four-arm Holliday-junctions with complementary sticky ends selfassemble into 2D DNA-arrays (reprinted with permission from ref. 6, copyright 2009, American Chemical Society). (B) DNA-origami using a 7 kb ssDNA template (black) that folds into a predesigned shape directed by ∼200 short “staple” strands. Cross-overs of the staple strands between helices provide rigidity to the structure. By computer aided design of the staple strand sequences the template strand can be folded in virtually any structure. Figure adapted from ref. 7 by permission from Macmillan Publishers Ltd: *Nature*, copyright (2006). (C) Predesigned DNA bricks selfassemble into a 3D cube. Each brick is formed by a 32 nt long oligonucleotide containing four domains that hybridize to neighbouring bricks. Any 3D shape can be obtained by omitting specific bricks from the cube, making this approach highly modular. Figure adapted from ref. 10. Reprinted with permission from AAAS.

In the early 80s, Seeman was the first to recognize that the well-defined spatial dimensions and predictable Watson–Crick base pairing render DNA an attractive building block for the self-assembly of nanoscale biomolecular structures.^5,6^ His approach was based on a four-arm Holliday-junction, formed by four single stranded oligonucleotides. Sticky ends on the four arms facilitated the sequence dependent assembly of the Holliday-junction subunits into higher order structures by sequence complementarity in the sticky ends (Fig. 2A). Subsequent ligation of the formed nicks created stable arrays. This generic bottom-up approach allowed the assembly of many different structures in a highly predictable manner. However, using exclusively short oligonucleotides was found to limit the scalability and complexity of the formed structures due to internal errors in the assembly process.

In 2006, Rothemund introduced a new approach to generate self-assembled, nanoscale structures from DNA.^7^ In this “DNA-origami” approach, one long single-stranded DNA template (7249 nucleotides, derived from the M13-phage) serves as a scaffold. The scaffold strand is folded and held in place in a predefined shape by hybridization with more than 200 so-called staple strands, small oligonucleotides obtained by computer aided design. Over the years, Rothemund's DNA-origami approach evolved from generating 2D “smiley faces” to complex 3D structures (Fig. 2B).^8,9^ Another strategy to construct complex three-dimensional DNA structures was recently developed by Yin and coworkers.^10,11^ Instead of one long template strand held in place by hundreds of staple strands, their approach makes use of DNA bricks. These small oligonucleotides comprise 4 domains of 8 nucleotides that can hybridize to four neighboring strands. Together, hundreds of these predefined bricks self-assemble into a molecular cube. By omitting specific bricks from the cube any three-dimensional shape can be readily obtained without redesigning individual sequences, making this approach highly modular (Fig. 2C).

**Fig. 3:**
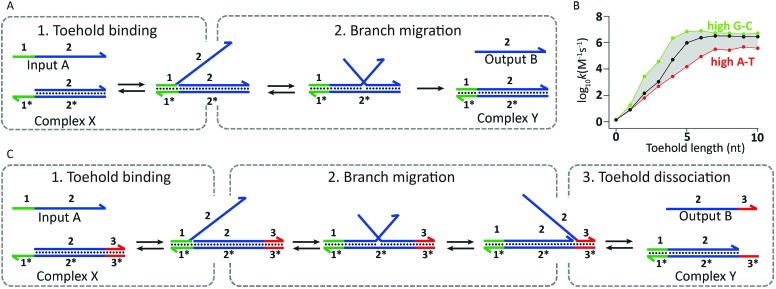
Underlying mechanisms used in dynamic DNA circuits. (A) Schematic representation of the toehold-mediated strand displacement reaction. Input A binds to the toehold-sequence 1* present on complex X, initiating subsequent branch migration. Completion of branch migration results in the release of output B and the formation of complex Y. Since the resulting complex Y has no available toehold, the reverse reaction is effectively not taking place. (B) The rate of toehold-mediated strand displacement is highly dependent on the length and sequence of the toehold domain. Increasing the stability of the toehold results in an enhanced displacement rate (adapted with permission from ref. 21, copyright 2009, American Chemical Society). (C) Schematic representation of the toehold-exchange mechanism. Input A binds to the toehold-sequence 1* present on complex X, initiating subsequent branch migration. After branch migration output B is bound to the complex *via* toehold-sequence 3*, which has to spontaneously dissociate for the toehold exchange reaction to complete, resulting in the release of output B and the formation of complex Y. Since complex Y contains a toehold-sequence to which output B can bind, the reverse reaction can also take place.

In addition to its attractive structural properties, networks of dynamically interacting DNA strands have allowed the development of DNA-based molecular computing.^12^ Examples range from circuits able to perform mathematical calculations,^13^ signal amplification,^14^ mimic small neural networks,^15^ solve Hamiltonian path problems^16^ and play molecular tic-tac-toe,^17^ to molecular robots and machines.^18^ The flow of information in these systems is often mediated by a mechanism known as toehold-mediated strand displacement. In toehold-mediated strand displacement a complementary single-stranded domain (the toehold) enables two DNA reactants to hybridize. Followed by this colocalization the incumbent strand is displaced by the invading strand *via* branch migration (Fig. 3A). The branch migration step is described as an enthalpic (hybridization) and entropic (strand release) driven random walk process and is non-directional.^18–20^ Zhang and Winfree determined the relation between the thermodynamics of toehold hybridization and the kinetics of toehold-mediated strand displacement. They showed that the overall rate of toehold-mediated strand displacement can be controlled over 6 orders of magnitude by changing the affinity of the toehold interaction (*i.e.* increasing the length and/or G–C content). Increasing the stability of the toehold increases the overall kinetics of toehold-mediated strand displacement (Fig. 3B).^21^ Although useful in some applications, a strong coupling between kinetics and thermodynamics (reaction speed and stability of the formed duplex) is undesirable for cascaded reactions in DNA-based molecular computing. To this end, Zhang and coworkers developed the toehold exchange reaction, as a more controllable version of toehold-mediated strand displacement. Similar to toehold-mediated strand displacement, the invading strand binds to a toehold, initiating branch migration and displacing the incumbent strand. However, the incumbent strand possesses a toehold that has to dissociate spontaneously to complete the reaction (Fig. 3C). The difference in base pairs formed by the invading and incumbent strand determines the direction of the reaction, whilst the rate of the overall reaction can be controlled by tuning the stability of the two toeholds. This uncoupling of thermodynamics and kinetics allows more control, enabling the construction of reaction networks that are entropically driven instead of enthalpy driven. In addition, the revealed toehold can be used in subsequent exchange reactions, which has been successfully employed in the construction of enzyme-free catalytic circuits.^22^ A notable example of using autonomous DNA-based logic circuits in biomedicine was reported by the group of Stojanovic.^23^ Using strand displacement cascades templated by oligonucleotide-conjugated antibodies, specific labeling was achieved of lymphocytes that expressed combinations of surface receptors such as CD45 and CD20. Extensive efforts have been devoted to regulate gene expression using toehold-mediated strand displacement reactions. Green *et al.* used toehold switches to block the ribosome binding site in RNA templates. By the addition of an RNA trigger partially complementary to the toehold switch, gene regulation could be switched on, allowing the construction of multi-input logic circuits.^24,25^

Having shown the seemingly endless possibilities of using DNA as a building block for the construction of nano-objects in almost any shape imaginable and the establishment of molecular computational networks of increasing complexity, the field of DNA nanotechnology is now increasingly focusing on potential applications in areas ranging from nanoelectronics^26^ to biology and nanomedicine.^27^ For the latter to become successful, generic strategies are required to interface these 3D nanostructures and DNA-based molecular computers to protein activity.

## 3. DNA as a template for protein assembly

An extensively used strategy to control protein activity is to use DNA as a template for colocalization of protein domains. The resulting increase in effective concentration can either promote the assembly of protein–protein complexes or enhance the efficiency of enzymatic reactions. Two approaches can be distinguished to allow the proteins of interest to bind to a specific DNA strand: (1) semisynthetic protein–DNA hybrids where proteins are covalently ligated to a single-stranded oligonucleotide and (2) expression of the proteins of interest in a fusion construct with a DNA-binding protein. Below, several well-established examples of each of these approaches will be discussed.

### 3.1 DNA-directed assembly of semisynthetic DNA–protein hybrids

**Fig. 4:**
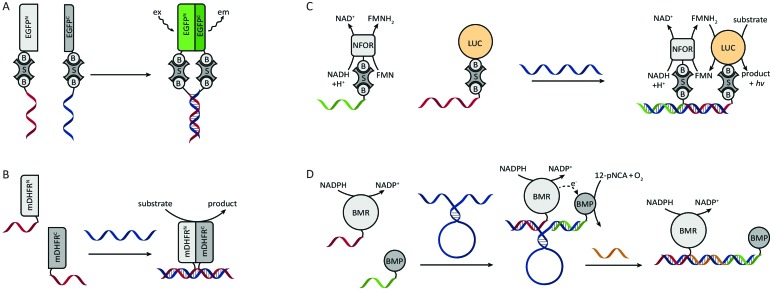
DNA-directed control of protein assembly *via* semisynthetic protein–DNA hybrids. (A) Split-EGFP complementation by DNA hybridization. Both biotinylated halves of split-EGFP are conjugated to two biotinylated oligonucleotides using streptavidin as crosslinker. Figure adapted from ref. 28. (B) DNA-directed complementation of split-mDHFR. Both protein halves are conjugated to a single-stranded oligonucleotide. Upon addition of a template strand that is complementary to the split-protein–DNA hybrids the protein halves colocalize and reassemble. Figure adapted from ref. 30. (C) DNA-templated assembly of a multi-enzyme complex. The biotinylated enzymes are conjugated to their biotinylated oligonucleotides using streptavidin as crosslinker. Efficient end product formation by the LUC luciferase is only observed upon colocalization of both enzymes on a shared template strand. NFOR reduces FMN to FMNH_2_, which is subsequently consumed by the neighbouring LUC to convert a substrate to product under the emission of a photon. Figure adapted from ref. 31. (D) DNA-templated reassembly of the two subdomains of cytochrome P450 BM3. Using a DNA scaffold that contains a stem-loop structure, the overall efficiency of the enzyme-cascade can be reversible controlled by hybridization to a complementary effector oligonucleotide (adapted with permission from ref. 34, copyright 2011, American Chemical Society).

One of the first examples of DNA-templated control of protein activity was the reassembly of split green fluorescent protein (EGFP) by Demidov *et al.* in 2006 (Fig. 4A).^28^ In this work each protein half was expressed with a terminal cysteine and subsequently biotinylated using a sulfhydryl-reactive reagent. Using the strong biotin–streptavidin interaction, complementary biotinylated oligonucleotides were connected to the split-EGFP halves. Upon mixing of the two split-EGFP–DNA hybrids the oligonucleotides hybridized, split-EGFP reassembled and consequently fluorescence rapidly increased (*t*_1/2_ ≤1 min). Similarly, Cissell *et al.* reassembled complementary DNA-split-luciferase hybrids, yielding a 15-fold increase in luminescence compared to non-functionalized split-luciferase.^29^ However, instead of using the biotin–streptavidin interaction for DNA functionalization, the split-luciferase halves were conjugated to thiol-modified oligonucleotides *via* an introduced cysteine and a bis-maleimide crosslinker. In the examples above a split protein was reassembled by direct interaction of two complementary oligonucleotides. To increase the modularity of these systems and allow them to respond to external DNA strands, Sancho Oltra *et al.* developed a split-murine dihydrofolate reductase (split-mDHFR) of which each half was conjugated *via* an inserted cysteine to a maleimide-functionalized oligonucleotide. Upon addition of a template DNA strand that is complementary to both oligonucleotide handles, the protein halves colocalized and reassembled into a functional enzyme (Fig. 4B).^30^ This modular, non-covalent approach allowed straightforward screening of template concentrations, showing an optimal substrate conversion at stoichiometric ratios of template and split-protein halves. In addition, a mismatch screen in the template strand revealed lower conversion rates as the number of mismatches increased.

All previous examples employed split-protein fragments for DNA-directed reassembly. Although split-proteins provide the advantage of low background signal, they usually suffer from thermodynamic instability. In addition, complementation is sometimes irreversible, *e.g.* in the case of split GFP where reassembly results in covalent bond formation during chromophore maturation. An alternative approach to split-proteins is to make use of multiple protein domains that complement each other's function. Niemeyer *et al.* recognized the advantage of using DNA-directed assembly for the generation of spatially ordered multi-enzyme complexes.^31^ From a catalysis point of view this formation of multi-enzyme complexes provides two benefits. First, reactions limited by diffusional transport of reaction intermediates are accelerated due to the proximity of the catalytic cores. Second, substrate channeling of reaction intermediates reduces the degree of side reactions, making the overall reaction more efficient. As a proof of principle, Niemeyer *et al.* developed an artificial multi-enzyme complex by colocalizing a biotinylated NADH:FMN oxidoreductase (NFOR) and a biotinylated luciferase (LUC) on a shared DNA template (Fig. 4C). Using NADH as an electron donor, NFOR reduces flavin mononucleotide (FMN) to FMNH_2_. Subsequently, LUC uses the FMNH_2_ and molecular oxygen to convert dodecanal to dodecanoic acid, while emitting a photon. A three-fold increase in overall catalytic activity was observed in the presence of the DNA-template compared to non-templated enzymes. The same principle of DNA-templated formation of multi-enzyme complexes was subsequently also employed for other enzyme cascades like glucose oxidase (GOx)/horseradish peroxidase (HRP)^32,33^ and cytochrome p450 MB3 subdomains.^34^ In the latter work, additional control was introduced by incorporating a stem loop structure in the template strand (Fig. 4D). Upon the addition of an effector oligonucleotide that is complementary to the loop sequence, the distance between the subdomains was increased. Consequently, the overall catalytic activity could be attenuated by varying the length of the effector oligonucleotide, *i.e.* the distance between the enzyme subdomains.

In addition to the organization of protein cascades on a one-dimensional DNA template, Wilner *et al.* used hexagon-like DNA strips that form highly defined two-dimensional planar structures.^35^ Using two-hexagon and four-hexagon wide strips two concatenated enzymes, GOx and HRP, were placed at specific distances from each other. Efficient formation of the final catalytic product was observed for the DNA templated enzyme cascade, whereas no product formation was observed in the absence of DNA template. The overall catalytic activity for the two-hexagon template was significantly higher than for the four-hexagon template, which was attributed to the larger distance between the concatenated enzymes in the four-hexagon template, leading to partial diffusion of intermediate products to the bulk solution. In addition to two concatenated enzymes, a similar distance dependency was observed between a cofactor (NAD^+^) and cofactor-dependent glucose dehydrogenase. This distance dependency was studied in more detail by Hao Yan and coworkers. To obtain even more defined distances between the GOx and HRP enzymes, they designed a two-dimensional DNA-origami tile as a planar scaffold, allowing interenzyme distances of 10 up to 65 nm.^36^ As expected, a clear distance dependency was observed, showing a decrease in overall catalytic activity upon increased interenzyme distances. More surprisingly was the unexpectedly high activity at an interenzyme distance of 10 nm, however, which was explained by the formation of a merged hydration layer around the proteins, providing a dimensionally-limited diffusion of H_2_O_2_ substrate between GOx and HRP.

### 3.2 DNA-templated assembly directed by DNA binding proteins

**Fig. 5:**
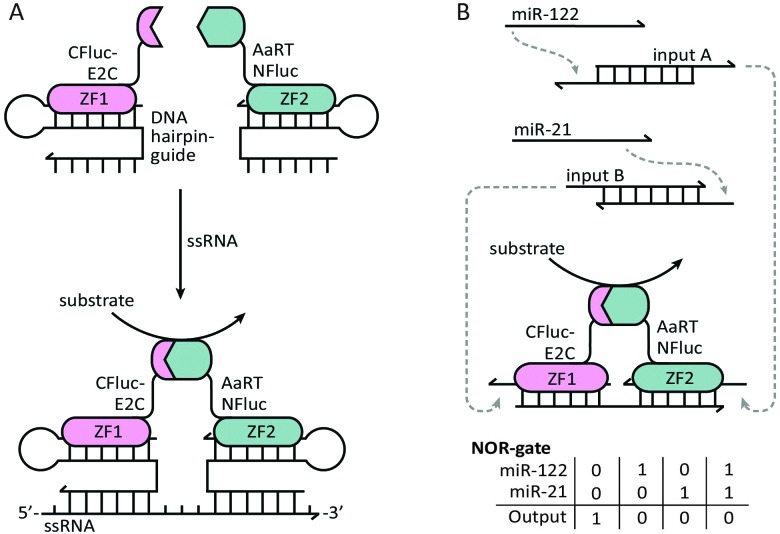
Controlling protein activity by zinc-finger mediated assembly on DNA/RNA templates. The two halves of a split-luciferase are fused to E2C and AaRT zinc-fingers, each recognizing a specific dsDNA duplex. (A) Both zinc-fingers bind to a dsDNA hairpin containing a ssDNA overhang. The hairpin functions as a guide to bind the zinc-finger, while the variable overhang facilitates hybridization with a ssRNA target sequence (reprinted with permission from ref. 40, copyright 2010, American Chemical Society). (B) NOR-gate functionality for the detection of two disease-related microRNAs. The presence of each microRNA induces a strand displacement reaction, resulting in the release of an oligonucleotide that can displace the template sequence from one of the two zinc-finger recognition motifs. Displacement from the template strand results in the disruption of the complemented split-luciferase and a decrease in luminescence. Figure adapted from ref. 41.

Ghosh and coworkers were the first to employ DNA binding proteins for the reassembly of split-proteins by introducing a method named sequence enabled reassembly of proteins (SEER). In this approach two non-fluorescent halves of a split-GFP were fused to two zinc fingers, each with a low nanomolar affinity towards a specific DNA sequence of 9 base pairs.^37^ Upon addition of a DNA duplex containing both recognition sequences, the split-GFP fragments reassembled, yielding a 7-fold increase in fluorescence. As expected, overall fluorescence decreased as the DNA spacer length increased. Besides the distance between the binding sites, the reassembly of the protein parts was also affected by their relative orientation. Local maxima in activity were observed every tenth base pair, showing that recovery of protein activity was optimal when both parts were aligned on the same side of the double helix. In addition to split-GFP, also split-β-lactamase was reassembled by SEER, resulting in a 1000-fold increase in enzyme activity upon DNA binding.^38^ By exchanging one zinc finger for a methyl-CpG binding domain that binds specifically to methylated DNA, SEER was also employed for the detection of methylated DNA as a potential marker for cancer.^39^ To increase the modularity of the SEER system, two hairpin DNA sequences were designed to be recognized by the zinc fingers. Extending the DNA hairpins with single stranded overhangs complementary to the target RNA enabled the zinc finger mediated reassembly of split-luciferase without any sequence restrictions (Fig. 5A).^40^ Recently, Deiters and coworkers used a similar strategy for the construction of various AND, OR and NOR logic operations, using split-luciferase complementation as readout. Depending on the type of gate, luminescence could be switched on or off by different combinations of two input oligonucleotides. As a proof of principle, a NOR logic gate was constructed that allowed the detection of two disease related microRNAs (miR-122 and miR-21). To this end, a toehold-mediated strand displacement step was incorporated to translate the microRNA sequences to oligonucleotides recognized by the two zinc-fingers (input A and input B). Subsequently, single stranded overhangs allowed each input to displace a zinc-finger recognition motif from the template strand, resulting in the disruption of complemented split-luciferase and a decrease in luminescence (Fig. 5B).^41^ An advantage of using sequence-specific DNA-binding domains such as zinc fingers instead of relying on oligonucleotide-conjugation is that these fusion proteins can be obtained by recombinant expression. Moreover, using genetically encoded components paves the way for DNA-templated control of protein activity *in vivo*.

## 4. Mechanical control of protein activity

A second molecular principle to control protein activity is taking advantage of the large difference in mechanical properties between single- and double-stranded DNA. As mentioned earlier, single-stranded DNA is a flexible polymer chain, whereas double-stranded DNA behaves like a rigid rod, at least at the 5–10 nm scale typical of protein (complexes). Seitz and coworkers employed the rigidity of dsDNA to control the activity of Src-kinase by modulating the affinity of a Src-kinase inhibiting phosphopeptide.^42^ To this end, a phosphopeptide–PNA chimera was developed that was confined in a non-binding loop conformation upon hybridization to a complementary DNA strand. Switching to a more extended, flexible conformation was achieved by displacement of the DNA strand by a specific RNA input strand, allowing the peptide to bind and inhibit Src-kinase. Below, several other strategies that have been developed to control the activity of proteins by switching between single- and double-stranded states of DNA are described.

### 4.1 Allosteric control of protein activity through DNA-springs

**Fig. 6:**
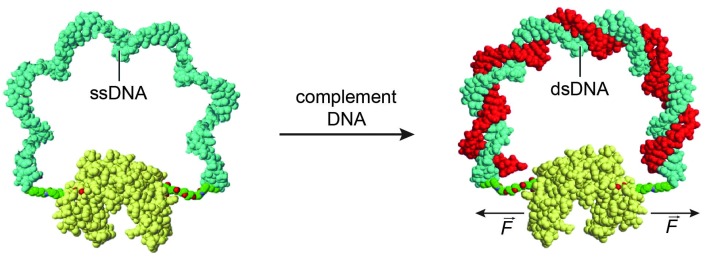
A mechano-sensitive enzyme actuated by a “DNA spring”. A 60 nucleotide long ssDNA linker is conjugated with both ends to the two lobes of guanylate kinase. By binding of the complementary oligonucleotide, the mechanical properties of the DNA linker change, transforming it into a rigid rod. The rigidity of the linker exerts a mechanical force on the protein, reducing its catalytic activity. Figure adapted from ref. 46. Copyright (2009), with permission from Elsevier.

Allosteric control of protein activity by ligand-induced conformational changes plays a pivotal role in key cellular processes including signal transduction, transcriptional regulation and metabolic control.^43^ Zocchi and coworkers pioneered an approach to install similar allosteric control of protein activity using DNA hybridization. In a first proof of principle study a maltose-binding protein (MBP)–DNA hybrid was created in which the two lobes of the protein were connected *via* a 60 nucleotides long ssDNA linker.^44^ Hybridization of a complementary oligonucleotide increased the mechanical tension on the protein, decreasing the binding affinity for maltose by 60%. The same strategy was employed to control the activity of two enzymes, Renilla luciferase and guanylate kinase (Fig. 6).^44–46^ In both cases DNA binding induced a decrease in enzyme activity, but DNA hybridization can also induce an increase in protein activity. Hybridization of a complementary oligonucleotide to ssDNA-constrained protein kinase A resulted in a 1.5 fold increase in PKA activity.^47^ These examples show that while allosteric control is a generic mechanism to regulate protein activity, the changes in activity are relatively modest and, because of its reliance on subtle conformational changes, not easily predicted. In addition, site-specific conjugation of a ssDNA linker between two attachments positions on the same protein can be synthetically challenging, resulting in low yields and the formation of non-responsive side products.

### 4.2 Mechanical control of enzyme–inhibitor complex formation

**Fig. 7:**
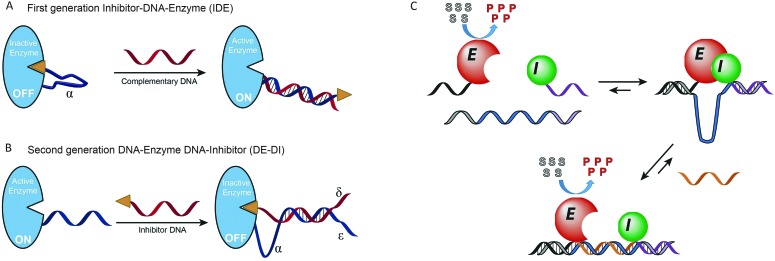
Mechanical control of enzyme–inhibitor complex formation. (A) First generation inhibitor–DNA–enzyme (IDE) system. A small-molecule phosphoramidite inhibitor is conjugated to the Cereus neutral protease enzyme *via* a ssDNA tether, allowing the inhibitor to bind to the enzyme intramolecularly. Hybridization to the complementary target DNA forces the inhibitor away from the enzyme (adapted with permission from ref. 48, copyright 2003, American Chemical Society). (B) Second generation DNA–enzyme–DNA–inhibitor (DE–DI) system. Hybridization of a DNA strand functionalized with a small-molecule inhibitor to an enzyme–DNA chimera. Multiple single-stranded regions (*α*, *δ* and *ε*) allow more complex operations like OR, NOR and AND Boolean logic gates. Figure adapted from ref. 49, copyright 2007, John Wiley and Sons. (C) Non-covalent formation of an enzyme–inhibitor complex *via* DNA tethers conjugated to both proteins. Due to their low intrinsic affinity TEM1-β-lactamase and the inhibitor protein BLIP only form an inactive complex upon binding to a template oligonucleotide that is complementary to both tethers. Enzyme–inhibitor complexation leads to the formation of a single stranded target recognition loop in the template strand. Binding of the complementary target oligonucleotide to this loop results in the disruption of the enzyme–inhibitor complex, reactivating enzyme activity (reprinted with permission from ref. 50, copyright 2015, American Chemical Society).

Instead of modulating protein activity by exerting a mechanical force on the protein itself, protein activity can also be controlled by mechanical control over the interaction between the protein and an inhibitor. In 2003 Ghadiri and coworkers were the first to employ DNA as an actuator of protein activity *via* mechanical disruption of an enzyme–inhibitor complex (Fig. 7A). For this, a 24 nucleotide long ssDNA linker containing a small-molecule phosphoramidite inhibitor was conjugated to Cereus neutral protease (CNP).^48^ The ssDNA tether allowed the small-molecule inhibitor to bind intramolecularly to the protein's active site, rendering the enzyme in its inactive state. Hybridization of a complementary target strand to the ssDNA tether resulted in the formation of a rigid double helix, which induced the physical separation of the small-molecule inhibitor from the enzyme, allowing the system to respond to concentrations of complementary target as low as 10 pM. To implement more complex signal processing possibilities, the system was redesigned by attaching a single stranded handle to the enzyme (Fig. 7B). Subsequently, a partially complementary inhibitor–DNA chimera was hybridized to the ssDNA handle, leaving three single-stranded regions on both the enzyme and inhibitor strand.^49^ These single-stranded regions could be used to program downstream Boolean logic operations like OR, NOR and AND gates. Despite the ease of incorporation of functional logic-gated architectures, in this case each individual target required the synthesis and purification of new DNA–protein hybrids. To facilitate system optimization and high throughput applications, our group developed a more modular approach to reversibly control enzyme–inhibitor complex formation (Fig. 7C).^50^ To this end, TEM1-β-lactamase and its inhibitor protein BLIP were each conjugated to a 21 nucleotide long tether. Due to their low micromolar affinity both protein domains only form an inactive enzyme–inhibitor complex upon hybridization to a template oligonucleotide complementary to both DNA tethers. The interaction between the enzyme and inhibitor results in the formation of a single-stranded loop region in the template strand, which is used as a target recognition loop. Upon hybridization of the target oligonucleotide to the template loop, the formed double helix disrupts the enzyme–inhibitor complex, and consequently reactivates the enzyme. The enzyme activity was found to depend on the length of the loop and target sequences. A systematic screening of loop and target strands ranging from 10 to 50 nucleotides in length, revealed that full restoration of enzyme activity required the formation of a loop-target helix of at least 40 base pairs. The modularity of the approach allowed easy exchange of the target recognition loop for any sequence of interest of at least 40 nucleotides long. Using a set of 8 viral DNA sequences, enzyme activation was only observed in the presence of the complementary target sequence, with a sensitivity as low as 10 pM. Finally, flanking the target sequence with a 10 nucleotide long toehold allowed multiple cycles of on and off switching upon the addition of fully complementary displacer strands.

### 4.3 DNA-tweezers to reversibly regulate protein activity

**Fig. 8:**
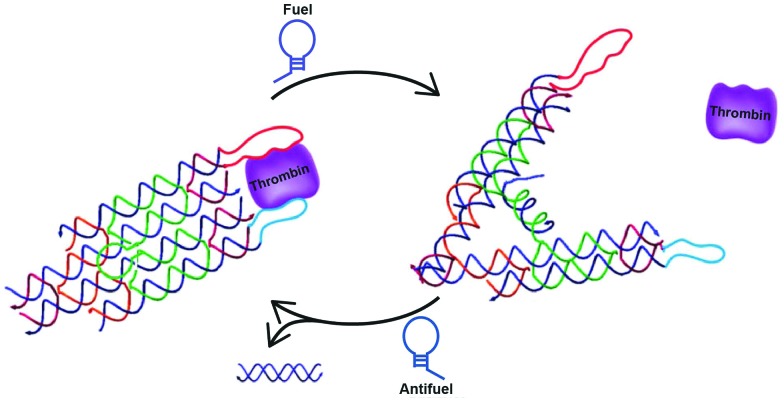
Reversible control of protein activity using DNA tweezers. Two double-crossover motifs are joined *via* a Holliday junction. The addition of a fuel strand, complementary to an internal stem-loop structure, induces the opening of the tweezer. Consequently, the distance between the tips is increased. Functionalization of the tips with specific aptamers allows switching between mono- and bivalent thrombin binding, reversibly releasing and capturing thrombin, respectively (reprinted with permission from ref. 51, copyright 2012, American Chemical Society).

A third, sophisticated way to precisely and dynamically control the distance between protein domains is the use of DNA tweezers. DNA-tweezers consist of two DNA double-crossover (DX) motifs that are joined *via* a Holliday junction. An internal stem-loop structure introduces dynamic properties that allows switching between open and closed conformation upon addition of fuel and antifuel strands, respectively. Making use of the extremely rigid DX motifs proved to be particularly useful, since the small switchable distance of the stem loop moiety is translated into large distances between the tips of the DX motifs. The first DNA tweezer that was reported allowed control of the distance between two thrombin binding aptamers, switching between weak monovalent binding and strong bivalent binding, hence releasing and capturing thrombin respectively (Fig. 8).^51^ Shortly after, DNA tweezers were employed in controlling the distance between the previously described enzyme cascade containing GOx and HRP, as well as the enzyme-cofactor pair GDH and NAD^+^, thus controlling the overall efficiency of the catalytic cascades.^52,53^ The development of these highly controllable chemical capture/release probes and chemical amplifiers provides promising tools for molecular diagnostics and intelligent materials.

## 5. Multivalent control of protein activity

Multivalent binding, where multiple low affinity interactions together yield a strong and highly specific interaction, plays a key role in biological processes ranging from cell–cell and cell–matrix interactions, the immune system, receptor clustering and protein–protein interactions.^54,55^ The importance of multivalency in nature renders multivalent ligands attractive as both sensors and actuators of biological processes. Most semi-synthetic multivalent ligands developed to date employ flexible templates that allow them to adopt to the precise orientation of their target, but at the cost of entropy. Using a rigid scaffold enhances the affinity and specificity of a multivalent interaction by decreasing the entropic penalty of binding. However, a rigid scaffold requires accurate spacing between binding moieties, as mismatched distances reduce the enthalpy of the interaction.^56^ DNA provides an attractive construction material for multivalent ligands, because of the ability to precisely control the spacing and orientation of ligands and tune the flexibility/rigidity of the scaffold. The self-assembling nature of DNA also allows rapid screening of many different template architectures.

### 5.1 Multivalent ligand presentation on a DNA scaffold

**Fig. 9:**
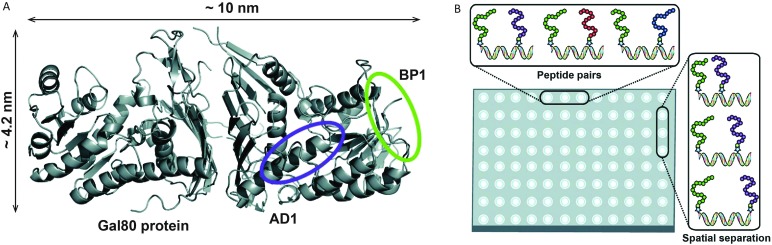
DNA as a scaffold for multivalent ligand display and subsequent strong protein binding. (A) As a proof of principle a synbody was created that binds multiple, non-overlapping regions of the yeast regulatory protein Gal80. (B) Combinatorial screening of multiple peptide ligands, as well as linker lengths and orientations on a high throughput surface plasmon resonance (SPR) chip allows easy screening for optimal peptide combination and inter-peptide distance (reprinted with permission from ref. 61, copyright 2009, American Chemical Society).

Several studies have used DNA as a linker to display multivalent ligands for protein binding with enhanced affinity and specificity.^57–60^ Using a combinatorial approach, Chaput and coworkers developed a general method to develop synbody constructs, displaying two peptides on a rigid dsDNA scaffold.^61^ As a proof of principle they showed a 1000-fold increase in affinity for a synbody containing two peptides that bind to the yeast regulatory protein Gal80 (Fig. 9). In a follow up study the same approach was used to generate a synbody against the growth factor receptor bound protein 2 (Grb2). Impressively, the developed synbody showed a five- to ten-fold stronger binding to Grb2 compared to commercial antibodies.^62^

**Fig. 10:**
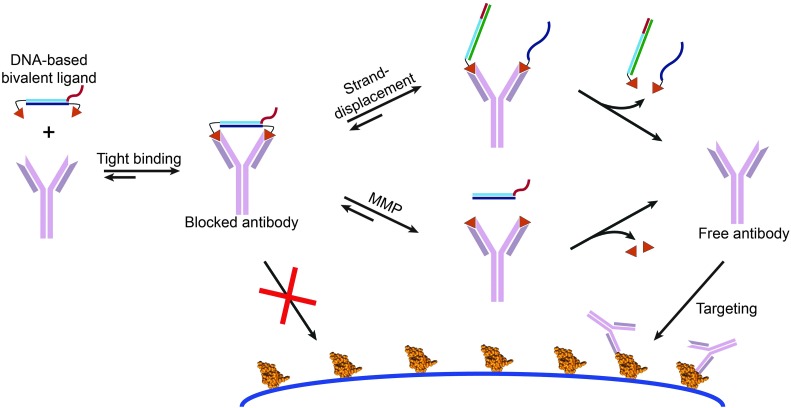
Bivalent peptide–DNA conjugates provide non-covalent and easily applicable molecular locks that allow the control of antibody activity using toehold-mediated strand displacement and/or protease activity. The rigidity of the dsDNA linker allows efficient bridging of the distance between the two antigen binding sites of the antibody of interest, resulting in a low entropic penalty and therefore tight bivalent binding. This blocks the antibodies antigen binding sites, prohibiting it to bind to its target antigen. The bivalent character of the DNA-based antibody ligand can be disrupted *via* strand displacement reactions using toeholds on the dsDNA blocker, or *via* matrix metalloprotease (MMP) mediated cleavage of a peptide linker between the epitope and dsDNA spacer. Disruption of the bivalent character results in a reduced affinity and dissociation of the monovalent, weak binding peptide epitopes, enabling the antibody to bind to its intended target. Figure adapted from ref. 65, copyright 2015, John Wiley and Sons.

These and other examples focused on developing efficient multivalent ligands, but in most cases did not employ these ligands to control protein activity.^63^ Our group recently introduced bivalent peptide–dsDNA ligands as effective, non-covalent and reversible antibody blockers. By conjugating a peptide epitope to both sides of a dsDNA spacer the relatively large distance between the antigen binding sites of the antibody is efficiently bridged, providing a low entropic penalty, *i.e.* strong bivalent antibody binding.^64^ Incorporation of a protease recognition sequence allowed cleavage of the linker between the peptide epitope and the dsDNA spacer, disrupting the bivalent interaction and releasing the antibody from the blocker. In addition, implementation of single stranded overhangs on the dsDNA spacer enabled activation of the antibody *via* YES, OR and AND logic-gated toehold exchange reactions.^65^ These bivalent peptide–DNA locks provide an opportunity to introduce additional specificity in antibody-based targeting by allowing matrix metalloprotease (MMP)-activity, the presence of miRNAs, or aptamer-activation to control antibody-based therapies, potentially resulting in less side effects (Fig. 10).

## 6. Aptamer based control of protein activity

One of the promises of DNA nanotechnology in biomedicine is its potential to create autonomous molecular systems that are able to interact with their environment, process these signals according to a predefined signal processing algorithm and translate the outcome of this molecular computing to control a specific biological process. The various approaches discussed in this perspective are well suited to translate the output of such systems to control protein activity. The first examples of such autonomous systems have recently been reported. Both examples use aptamers to sense their environment and translate the presence of a specific biomarker to an oligonucleotide input.^66–68^

**Fig. 11:**
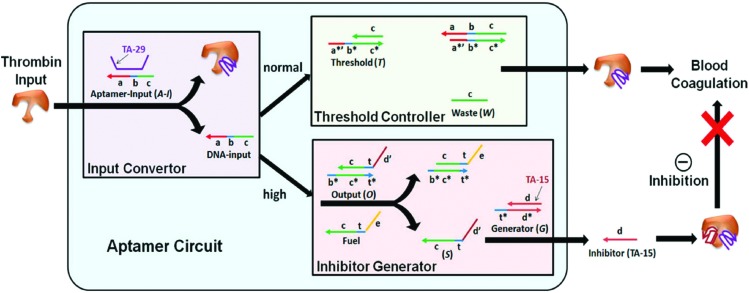
Concept of autonomously regulated control of thrombin protein concentration *in vitro*. The aptamer circuit consists of three modules; an input converter, where the thrombin input is converted *via* aptamer-binding to release an oligonucleotide, a threshold controller that captures the DNA input up to a predefined threshold concentration, and an inhibitor generator that releases an inhibiting thrombin aptamer only when the DNA input concentration exceeds the predefined threshold concentration (reprinted with permission from ref. 69, copyright 2012, American Chemical Society).

Weihong Tan and coworkers reported an autonomous system to control the concentration of thrombin (Fig. 11).^69^ Thrombin is a protease that initiates blood coagulation and whose concentration and activity in the bloodstream need to be carefully controlled. Their autonomous circuit was composed of an input converter that converts thrombin to a DNA signal, a threshold controller that defines the threshold concentration at which the protein concentration is maintained, and an inhibitor generator that inhibits protein concentrations above the predefined threshold value. Key for this application was the use of two anti-thrombin aptamers. One aptamer binds to the heparin exosite without exerting any inhibitory action (TA-29), therefore acting as the input converter. The second aptamer binds to the fibrinogen exosite (TA-15) and has a strong inhibiting effect on thrombin, therefore acting as the inhibitor generator module. Upon introduction of thrombin, the TA-29 aptamer binds to the protein while releasing an oligonucleotide. This oligonucleotide subsequently serves as an input for a cascade of toehold-exchange reactions. Once a predefined threshold concentration of input oligonucleotide is exceeded, a second, thermodynamically less favorable cascade is activated that leads to the release of the TA-15 aptamer. Hence, excessively high concentrations of thrombin are inhibited. Although the system operates autonomously, DNA-based cascade components are not recycled and continuous administration of the modules would be required for sustained performance of the system.

**Fig. 12:**
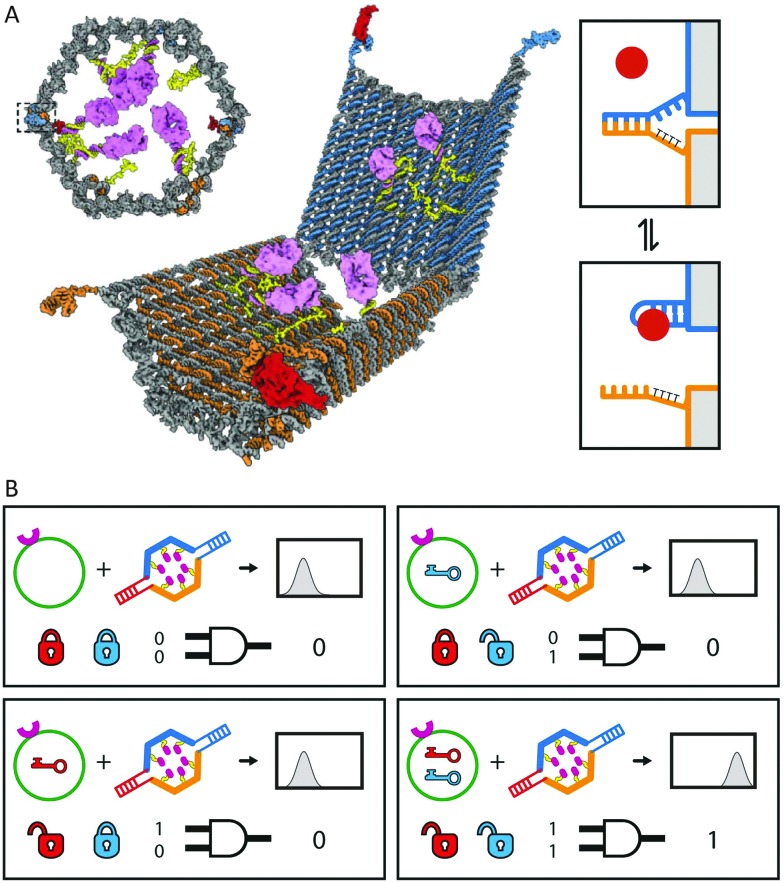
Autonomous cell surface evaluation by a DNA nanorobot carrying a single chain antibody fragment payload. (A) The hexagonal barrel is loaded with antibody fragments towards human leukocyte antigen and closed by two aptamer based locks, refraining the antibody fragments from binding to the cell surface target. (B) Only in the presence of the right combination of protein inputs the aptamer locks change conformation and allow the barrel to open. Consequently the encapsulated antibodies are able to bind to the cell surface. Figure adapted from ref. 27. Reprinted with permission from AAAS.

Another intriguing glimpse of the potential of autonomous DNA-based molecular systems is the work of Church and coworkers, who reported the construction of DNA-based nanocontainers to control the accessibility of cancer cell-targeting antibody fragments (Fig. 12).^27^ Their nanocontainers consist of a hexagonal barrel composed of two domains, intramolecularly connected *via* single-stranded hinges at the bottom. On top, aptamer-based locks keep the barrel in its closed conformation in absence of the correct combination of input proteins. Antibody payloads functionalized with an anti-handle are hybridized to handles displayed on the inner surface of the barrel, rendering the payload inaccessible to the nanocontainer's environment. When the correct combination of input proteins is present, the aptamers switch conformation allowing the barrel to open. Consequently, the payload is no longer shielded and can bind to cells displaying the correct antigen, *i.e.* the protein's activity is controlled by physically separating it from the environment. Using different combinations of aptamer locks, logic AND gates were created, increasing the selectivity towards the target cells. These DNA-based nanorobots have proven to successfully target specific cells in living insect models and are currently undergoing clinical trials.^70^

## 7. Conclusion and outlook

In this feature article we introduced three generic strategies to control protein activity using oligonucleotide-based triggers. The first approach employs DNA as a template for protein complex formation such as the reassembly of split proteins and the assembly of enzymatic cascades. Typically, systems based on cascade catalysis show more modest rate enhancements in the presence of a DNA template compared to split enzyme systems. However, the latter may suffer from limited thermodynamic stability. Moreover, the development of useful split variants of a target protein typically requires extensive protein engineering. A second, extensively used strategy is to exploit the difference in mechanical properties between single-stranded (flexible) and double-stranded DNA (rigid). Mechanical strain can be directly applied on a protein by linking two sites in a protein *via* a single stranded DNA linker, followed by hybridization to a complementary strand. As this method relies on direct allosteric control of protein dynamics, the optimal design is hard to predict and this approach generally results in a modest dynamic range. A more modular approach is to use DNA hybridization to disrupt the interaction between a protein and an inhibitor, which can be either a small molecule or a protein. A third, generic strategy uses multivalent interactions between a DNA-based multivalent ligand and a protein target. Modulation of protein activity can be achieved by switching between monovalent and bivalent architectures, or by tuning the structural properties of the ligand. An interesting example of the latter approach is control of antibody activity by bivalent peptide–DNA conjugates.

An important hurdle to overcome for successful applications of these and related strategies such as the encapsulation of proteins in DNA nanostructures *in vivo*, is the limited chemical stability of DNA in physiologically relevant matrices such as blood. Although the compact arrangement of DNA-based nanostructures may provide some protection against enzymatic degradation compared to simple ssDNA and dsDNA, recent studies have shown that this stability is still limited.^71^ A variety of chemical modifications of both the nucleobases and sugar-phosphate backbone have been reported to increase the hydrolytic stability of ssDNA and dsDNA, however.^72^ These include phosphorothioate modification of the backbone, the use of a peptide-based backbone in peptide nucleic acids (PNA) and conformational constraining of the backbone by introducing a methylene bridge between the 2′-O and 4′-C of the sugar in locked nucleic acids (LNA).^73–75^ Successful application of these chemically modified oligonucleotides will require a thorough understanding of the influence of these modifications on their structural properties as well as on the kinetics and thermodynamics of hybridization.^76,77^

Most of the examples described in this review, in particular those based on mechanical control and templated assembly of protein complexes, involved the use of protein–DNA hybrids obtained *via* covalent coupling of the protein with a synthetic oligonucleotide. The strength of this approach is that the properties of these systems can be rationally controlled by tuning the free energy of DNA-hybridization. While the development of new chemoselective bioconjugation methods has made the synthesis of protein–DNA conjugates more straightforward, purification of the conjugates from unreacted and/or excess reagents is often required. An alternative strategy is to genetically fuse the protein of interest to sequence-specific DNA-binding domains such as zinc finger proteins. In addition to avoiding the time-consuming synthesis of protein–DNA conjugates, this approach is particularly attractive when considering *in situ* applications of DNA/RNA-based control of protein activity. Despite extensive protein engineering efforts, however, controlling the DNA-binding affinity and specificity of zinc finger mediated strategies is still limited compared to approaches based on hybridization of complementary oligonucleotide strands. In this respect, the use of other modular DNA binding domains such as TALENs or RNA-guided CAS9-based strategies could be considered for future applications.^78^ The latter approach may be particularly attractive as it does not rely on protein-based sequence recognition, but make use of guide RNAs.^79^ Approaches to control the activity of proteins by multivalent presentation of protein binding ligands or binding *via* DNA-based aptamers are particularly attractive, since these non-covalent approaches do not rely on chemical or genetic modification of the target protein. Critical for such approaches is the availability of peptides, aptamers and other ligands that not only bind, but also block the activity of the protein.^80,81^

In this review we focussed on molecular approaches for DNA-based control of protein activity. However, for the construction of systems capable of autonomously diagnosing and treating diseases, sensing strategies that accept protein activity as input for DNA-based molecular networks are equally important. Most *in vitro* approaches to translate protein activity to an oligonucleotide signal are based on aptamers, but the number of proteins for which high affinity aptamers have been developed is limited. Many of the strategies discussed here are reversible, however, and could be redesigned to allow protein-based control of DNA-based molecular circuits. *E.g.* instead of controlling the activity of proteins by co-localizing them on a DNA template the opposite, where the assembly of oligonucleotides is triggered on a template protein, can be employed to translate the presence of a protein trigger to a DNA-based output.^82^ A well-known example is proximity-based ligation, in which DNA-hybridization is triggered by the antibody-mediated assembly of oligonucleotides on a protein-scaffold, subsequently serving as input to start a rolling circle amplification reaction.^83^ The development of these and other molecular strategies to integrate the rich functional properties of proteins with the inherent programmability of DNA–nanotechnology will provide access to truly autonomous biomolecular systems with sophisticated signal integration, processing and actuation properties.
